# The validity of a rheumatoid arthritis medical records-based index of severity compared with the DAS28

**DOI:** 10.1186/ar1921

**Published:** 2006-03-14

**Authors:** Masayo Sato, Sebastian Schneeweiss, Richard Scranton, Jeffrey N Katz, Michael E Weinblatt, Jerry Avorn, Gladys Ting, Nancy A Shadick, Daniel H Solomon

**Affiliations:** 1 Division of Pharmacoepidemiology, Department of Medicine, Brigham and Women's Hospital, Harvard Medical School, 1620 Tremont Street, Suite 3030, Boston, MA 02120, USA; 2 Boston VA Medical Center, 150 South Huntignton Avenue, Jamaica Plain, MA, 02130, USA; 3 Division of Rheumatology, Immunology and Allergy, Department of Medicine, Brigham and Women's Hospital, Harvard Medical School, 75 Francis Street, Boston, MA, 02115 USA

## Abstract

The objective of this work was to assess the convergent validity of a previously developed rheumatoid arthritis medical records-based index of severity (RARBIS) by comparing it with the 28-joint Disease Activity Score (DAS28). This study was conducted in subjects within the Brigham and Women's Hospital Rheumatoid Arthritis Sequential Study (BRASS). We selected 100 patients with rheumatoid arthritis (RA) from the BRASS with DAS28 scores equally distributed in four quartiles. The medical records were reviewed to calculate the RARBIS, which includes indicators from the following categories: prior surgical history, radiologic and laboratory findings, clinical and functional status, and extra-articular manifestations. The Spearman correlation between the RARBIS and the DAS28 was assessed in the total study population and in relevant subgroups. We re-weighted on subscales and recalculated the RARBIS score. This was performed based on findings of correlations between the DAS28 and subscales; and also the result from a multiple linear regression with the DAS28 (as a dependent variable) and five subscales (as independent variables). The mean RARBIS was 4.36 (range 0–11). Among the total study cohort, the RARBIS was moderately correlated with the DAS28 (*r *= 0.41, 95% confidence interval [CI] 0.23–0.56). In subgroup analyses, including age, gender, rheumatoid factor status, and disease duration, we found no statistically significant differences in the correlations. After re-weighting, the correlation between the RARBIS and the DAS28 was somewhat improved (*r *= 0.48, 95% CI 0.31–0.62). In conclusion, the RARBIS correlated moderately well with the DAS28 in this population. The RARBIS has both face and convergent validity for patients with RA and relevant subgroups and may have application for medical records studies in patients with RA.

## Introduction

Rheumatoid arthritis (RA) is a chronic inflammatory disease that can lead to long-term joint damage resulting in chronic pain, loss of function, and disability [1]. It causes substantial morbidity in most patients and premature mortality in many [2-5]. Some patients with severe RA may be at higher risk for complications such as infection, gastrointestinal problems, heart disease, and cancer [6,7]. However, those complications may be related to adverse effects of drug therapies rather than to the effect of RA.

Several studies have reported serious but rare adverse effects of RA medications. For example, biologic agents that block the action of tumor necrosis factor-α have been investigated as the cause of serious infections, hematological cancers, and demyelinating disease [8-11]. Lymphoproliferative malignancies among users of disease modifying anti-rheumatic drugs have also been reported [12,13].

Data on adverse drug events come predominantly from clinical trials, case reports, case series, and epidemiologic studies. Randomized clinical trials are limited in their ability to detect rare adverse effects because of small sample size, selection of patients least likely to experience toxicity, and short duration of follow-up. It is difficult to base causality assessment on case reports/series. Therefore, pharmacoepidemiologic studies can play a pivotal role in evaluating safety of medications used in RA. However, the severity of RA may affect the choice of medication and RA outcomes. Failing to control for RA severity in epidemiologic studies may lead to biased estimates of the association between RA drug treatment and RA outcomes. This type of bias, confounding by indication, is an important potential bias in many pharmacoepidemiologic studies [14,15].

To measure RA disease severity in medical records, we defined a set of indicators of severe RA through an expert Delphi panel of rheumatologists [16]. On the basis of their findings, we developed an RA medical records-based index of severity (RARBIS) [17]. The goal of this project was to test the convergent validity of the RARBIS with another accepted RA measure. However, there is no standard criteria for RA severity that can be assessed from medical records.

Although disease activity and disease severity are not synonymous, we decided the correlation of the RARBIS with the 28-joint Disease Activity Score (DAS28), a widely used instrument that affects treatment decisions by rheumatologists in daily clinical practice. The DAS28 is a statistically derived index consisting of number of swollen joints, number of tender joints, erythrocyte sedimentation rate (ESR), and general health [18,19]. Thus, recognizing the methodologic limitations of this approach, we chose to assess the convergent validity of the RARBIS by comparing it with the DAS28, a well accepted RA disease index.

## Materials and methods

### Study population

The Brigham and Women's Hospital Rheumatoid Arthritis Sequential Study (BRASS) is a cohort of patients with confirmed RA who receive care from rheumatologists in the Division of Rheumatology, Immunology and Allergy at our hospital. Every six months, patients are asked to complete questionnaires regarding general health information, medications for RA, health status, and surgical history for RA. Clinicians complete a similar questionnaire, including global assessment and joint counts. For our study, patients were identified from the BRASS cohort between March and October 2003. Identified patients during the study period were partitioned in quartiles based on their baseline DAS28 scores. We randomly selected 100 RA patients with the baseline DAS28 equally distributed in four quartiles.

## RARBIS

The RARBIS was developed through an expert Delphi panel of six rheumatologists, and convergent validity was assessed by comparing it with intensity of the actual RA treatments that patients received [16,17] (Table 1.) The index includes indicators from five categories: prior surgical history (C1–C2 fusion and joint surgeries), radiologic findings (C1–C2 subluxation and erosions), laboratory findings (rheumatoid factor status, ESR, C-reactive protein, and platelet counts), clinical and functional status (arthritis flares, morning stiffness, physician global rating, and functional status), and extra-articular manifestations (vasculitis and pulmonary nodule). These indicators are available in medical records. The total RARBIS score was calculated by summing the five subscales. Because joint destructions and surgical histories were considered by the Delphi panel to be indicators strongly associated with severe RA, the prior surgical and radiologic subscales were given higher weights in the total RARBIS score. The surgical subscale and radiologic subscale account for 60% of the total RARBIS score. The RARBIS score with medication use was also calculated with the addition of data on medication use.

### Data collection and processing

Electronic medical records were reviewed to collect information on demographic characteristics, clinical and functional status, laboratory test results, and radiology reports. Data on clinical status indicators and laboratory test results were collected for one year prior to baseline. If a patient's clinical and functional status changed during that period, the worst condition was recorded. When there was an expression indicating disease activity such as "flare up," "ongoing," and "active" in medical records, we counted it as having a flare. The number of flares in the last year was summed. If there was no information regarding clinical and functional status on the medical chart, we assumed a patient had no apparent clinical and functional manifestations during that period. The value associated with the most severe disease activity in the preceding year was recorded. Whether patients ever had any erosions and C1–C2 subluxation was examined in radiology reports. We obtained information on surgical history, extra-articular manifestations, physician's global assessment of arthritis activity, and medication use from a questionnaire at baseline in BRASS, which was completed by patients and physicians. If data were missing on radiology findings, laboratory tests, surgical history, or extra-articular manifestations, we assumed that no clinical information or radiologic/laboratory reports suggested that patients did not havesignificant RA symptoms at that time. The study was approved by the Institutional Review Board of Partners HealthCare (Boston, MA, USA).

### Statistical analysis

To test the convergent validity of RARBIS as a measure of disease severity, the Spearman rank correlation between the RARBIS and the DAS28 was assessed in the total study population and in relevant subgroups, including age, gender, rheumatoid factor status, and disease duration. To examine whether different subscale weights improved the performance of the index, we re-weighted subscales and recalculated the RARBIS score. The original weights of the RARBIS were determined based on the finding of the Delphi panel rating [16]. The surgical subscale was weighted more than the clinical subscale. The new weights were derived from the correlations between the DAS28 and subscales and the result from a multiple linear regression with the DAS28 as a dependent variable. Because clinical indicators and radiologic indicators were closely correlated with the DAS28 and the surgical history was not correlated with the DAS28, we up-weighted the clinical subscale and down-weighted the surgical subscale. Three different weighting systems included: exchanging the maximum scores of the clinical status subscale and the surgery subscale; up-weighting the clinical status subscale by a factor of two and removing the surgery subscale and the extra-articular manifestation subscale; multiplying each subscale by the regression coefficients of a linear regression of all subscales on the DAS28.

## Results

### Patients characteristics and the RARBIS score

**Figure 1:**
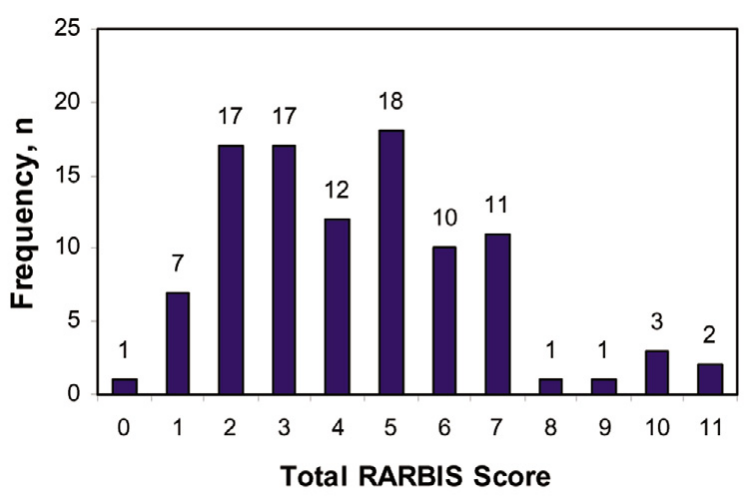
Frequency distribution of the rheumatoid arthritis medical records-based index of severity (RARBIS).

Patient characteristics of the study cohort are summarized in Table 2. The mean age of patients was 59 years with the majority less than 65 years. Most of the patients were female (81%) with mean disease duration of 16 years. The mean value of the DAS28 was 4.2 (standard deviation [SD] 1.6) at baseline. The mean total RARBIS score calculated for all patients was 4.36 (SD 2.37, range 0–11) (Figure 1). The distributions of each subscale and total score are shown in Table 3. The mean of the clinical subscale was 1.43 (SD 1.20) with a range of 0–3. Patients in the study had few surgeries, few extra-articular manifestations, and low scores on the radiology subscale.

### Comparisons of the RARBIS with the DAS28

**Figure 2:**
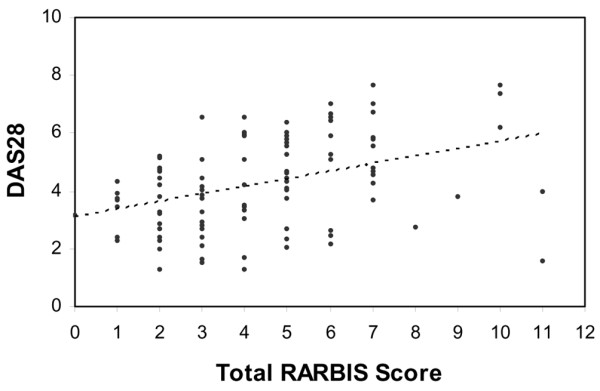
Scatter plot of the rheumatoid arthritis medical records-based index of severity (RARBIS) versus the 28-joint Disease Activity Score 28 (DAS28).

Table 4 shows the Spearman correlation with 95% confidence interval (CI) between the RARBIS and the DAS28. The total score was moderately correlated with the DAS28 (*r *= 0.41, 95% CI 0.23–0.56). Figure 2 shows scatter plots of the paired RARBIS scores and corresponding DAS28 scores. Two components of the total score, the clinical subscale and the X-ray subscale, were significantly related to the DAS28. The laboratory subscale had a weak correlation with the DAS28. The other components were not correlated with the DAS28. When the subscale of medication use was added to the RARBIS, the score was also significantly correlated with the DAS28, though the correlation was attenuated.

Correlations between the DAS28 and the RARBIS among subgroups are shown in Table 5. In subgroup analyses, we found no statistically significant differences between the subgroups in their correlations between the DAS28 and the RARBIS. However, the sample size in each subgroup was small.

### Re-weighting

Up-weighting on the clinical subscale and down-weighting on the surgical subscale slightly improved the correlation with the DAS28. Different weights did not make large differences in correlation between the RARBIS and the DAS28 (Table 6).

## Discussion

The results of our study provide support for the convergent validity of the RARBIS with the DAS28. We are hopeful that the RARBIS can be used to help define RA severity but recognize that there are no well accepted measures of RA severity, making validation quite a challenge. RA severity is considered a complex measure determined by objective components such as disease activity (for example, tender/swollen joints and acute-phase reactants) and physical damage (for example, radiologic damage, and functional disability) and subjective components (for example, global health assessment, pain, grip strength, fatigue, and costs) [20,21]. We chose to use the DAS28, a measure of disease activity, because it is commonly used for therapeutic decision-making.

Symmons and colleagues [22] developed a measure of overall status in RA composed of demographic details, activity score, damage score, and drug treatments [23]. Bardwell and colleagues [24] reported a brief measure of severity for RA based on physician's rating of disease activity, functional impairment, and physical damage. Navarro-Cano and colleagues [25] measured RA disease severity using an RA component of the Duke Severity of Illness Checklist through a physician's assessment. A measure of RA functional status based on American College of Rheumatology standards [26] involves clinical assessment (for example, physical examination), laboratory tests (for example, ESR), and imaging procedures (for example, X-rays and magnetic resonance imaging), which may be the most accurate measure of RA severity. Unlike those measures, the RARBIS does not require a new physician's assessment and the information on indicators can be routinely collected from typical medical records. Thus, the RARBIS may have great utility for researchers using medical records.

The present study has several limitations. As we fully recognize and have noted, because there is no standard RA severity measure, we assessed the convergent, and not the criterion, validity of our index by comparing it with the DAS28. We assumed that the DAS28 should be highly correlated with RA severity because disease activity is deemed one of the core determinants of RA disease severity and the DAS28 includes patient-reported disease status. To enhance information for the RARBIS, we obtained information on surgical history, extra-articular manifestations, and physician's global assessment of arthritis activity from a questionnaire at baseline in BRASS. This modification did not change the properties of the index score in the present study.

## Conclusion

We have examined the convergent validity of a medical records-based index of RA severity, the RARBIS. It is obtained simply by forming an arithmetic sum of indicators on a medical record. We propose it as a practical and useful tool for measuring RA severity. Because the RARBIS does not require a physician's assessment for its calculation, it is easily applied retrospectively to medical records. All of the data for the RARBIS are accessible from medical records. The ease of collecting these data is greatly enhanced by an integrated medical record that contains all laboratory, radiologic, and surgical information. In studies that assess associations between treatments and RA outcomes, the RARBIS will be useful to control for confounding by RA severity. Before our index can be recommended, it would be beneficial to further evaluate it in other populations as well as use other gold standards.

## Abbreviations

BRASS = Brigham and Women's Hospital Rheumatoid Arthritis Sequential Study; CI = confidence interval; DAS28 = 28-joint Disease Activity Score; ESR = erythrocyte sedimentation rate; RA = rheumatoid arthritis; RARBIS = rheumatoid arthritis medical records-based index of severity; SD = standard deviation.

## Competing interests

The authors declare that they have no competing interests.

## Authors' contributions

MS collected and analyzed data and drafted the manuscript. SS provided statistical support and helped edit the manuscript. RS provided data access and helped edit the manuscript. JNK contributed conceptual advice and helped edit the manuscript. MEW provided data access and conceptual advice and helped edit the manuscript. JA contributed conceptual advice and helped edit the manuscript. GT collected data and helped edit the manuscript. NAS provided data access and helped edit the manuscript. DHS provided conceptual design, analytic support, and data access and helped edit the manuscript. All authors read and approved the final manuscript.

## Figures and Tables

**Table 1:** Rheumatoid arthritis severity index

**Subscale**	**Points**
**Surgery subscale:**	
C1–C2 fusion	3 points
Any hand joint	1 point
Any foot joint	1 point
Major joints	1 point each
(hips, knees, shoulder, elbow, wrist, ankle)	(maximum of 2)
Maximum score for category:	**5 points**
**X-ray subscale:**	
C1–C2 subluxation	3 points
Any erosions	1 point
Maximum score for category:	**4 points**
**Extra-articular manifestations subscale:**	
Vasculitis	1 point
Pulmonary lung nodule	1 point
Maximum score for category:	**1 point**
**Clinical status subscale:**	
Arthritis flares	
1	1 point
2–4	2 points
5+	3 points
Worst physician global rating: "doing poor"	2 points
Functional status	
Unable to do hobbies	1 point
Unable to work	2 points
Unable to care for self	3 points
Hours of morning stiffness <1	0 points

Summed points in each category must not exceed the maximum score for the category. For example, a patient could have 10 points in the clinical status subscale but would be assigned the maximum for that category (3 points).

**Table 2:** Characteristics of patients with rheumatoid arthritis (*n *= 100)

Characteristics	Number of Patients
Age, mean (SD (range)), years	59 (13.3 (25–86))
Age diagnosed for RA, mean (SD (range)), years	43 (14.3 (16–78))
Duration of RA, mean (SD (range)), years	15 (11.9 (0–50))
Number of rheumatology visits in last year, mean (SD (range))	4.5 (2.0 (1–12))
DAS28, mean (SD (range))	4.2 (1.6 (1.3–7.7))
Female gender	81 (81)
Highest level of education: > high school	96 (96)
Physician global rating: poor	11 (11)
Hospitalized for RA in last year: yes	3 (3)
ACR Functional Classification* (worst in last year)	
Class I (no limitation)	33 (33)
Class II (self-care, working, no hobbies)	2 (2)
Class III (self-care, not working, no hobbies)	5 (5)
Class IV (limited self-care, bed-bound)	6 (6)
Hours of morning stiffness^† ^(worst in last year), hr	
< 1	73 (73)
1 to 4	23 (23)
> 4	4 (4)
Number of flares in last year^‡^	
0	38 (38)
1	30 (30)
1 to 4	29 (29)
> 5	3 (3)
Pulmonary nodule^¶^: ever	5 (5)
Vasculitis: ever	0 (0)
Joint erosion^¶^: ever	70 (70)

*Fifty-four patients had undefined ACR Functional Classification based on chart review.

^†^No record of morning stiffness in the medical record was counted as <1 hour.

^‡^No record of flares in the medical record was counted as no flares (zero).

^¶ ^Missing data were assumed to be the absence of the extra-articular manifestation for pulmonary nodule. When radiologic and laboratory data were not available, the data was considered as missing. ACR, American College of Rheumatology; DAS28, 28-joint Disease Activity Score; RA, rheumatoid arthritis; SD, standard deviation.

**Table 3:** Distributions of subscales and total scores

	Possible maximum	Mean (SD)	Observed minimum	Median	Observed maximum
Total RARBIS score	15	4.36 (2.37)	0	4.00	11
Subscale:					
Clinical	3	1.43 (1.20)	0	1.00	3
Surgery	5	0.57 (1.00)	0	0.00	5
X-ray	4	1.00(1.07)	0	1.00	4
Extra-articular manifestations	1	0.05 (0.22)	0	0.00	1
Laboratory	2	1.31 (0.63)	0	1.00	2
Medication	3	2.00 (1.06)	0	2.00	3
Total score with medication	18	6.36 (2.73)	1	6.00	14

The total RARBIS score excludes the medication subscale and was calculated by summing the clinical, surgery, X-ray, extra-articular manifestation, and laboratory subscales. The observed minimum scores were zero for each subscale and the total RARBIS (without medications). However, when the medication subscale is included in the total score, we did not observe any patients with zero points. SD, standard deviation.

**Table 4:** Spearman's rank correlation of the total RARBIS score with the DAS28

Score	Spearman	95% CI	*p *value*
Total RARBIS score	0.41	0.23 – 0.56	<0.0001
Subscales:			
Clinical subscale	0.42	0.24 – 0.57	<0.0001
Surgery subscale	0.09	-0.11 – 0.28	0.3690
X-ray subscale	0.34	0.15 – 0.50	0.0005
Extra-articluar subscale	0.002	-0.19 – 0.20	0.9875
Laboratory subscale	0.12	-0.09 – 0.30	0.2890
Medication subscale	-0.01	-0.21 – 0.18	0.8860
Total score with medication	0.33	0.14 – 0.49	0.0007

* *p *value for correlations

The total RARBIS score excludes the medication subscale and was calculated by summing the clinical, surgery, X-ray, extra-articular manifestation, and laboratory subscales. CI, confidence interval; DAS28, 28-joint Disease Activity Score; RARBIS, rheumatoid arthritis medical records-based index of severity.

**Table 5:** Correlations between the DAS28 and the total RARBIS score among subgroups

Subgroup	Spearman	95% CI	*p *value*
Age			0.51
< 65 years (*n *= 65)	0.45	0.23 – 0.63	
≥ 65 years (*n *= 35)	0.33	0.00 – 0.60	
Gender			0.40
Male (*n *= 19)	0.52	0.09 – 0.79	
Female (*n *= 81)	0.35	0.14 – 0.53	
Rheumatoid factor			0.69
Negative (*n *= 27)	0.51	0.16 – 0.75	
Positive (*n *= 67)	0.44	0.23 – 0.62	
Duration of RA			0.96
< 5 years (*n *= 19)	0.35	-0.13 – 0.69	
≥5 years (*n *= 81)	0.37	0.16 – 0.54	

* *p *value for differences between subgroup correlations

CI, confidence interval; DAS28, 28-joint Disease Activity Score; RA, rheumatoid arthritis; RARBIS, rheumatoid arthritis medical records-based index of severity.

**Table 6:** Correlations between the DAS28 and the revised RARBIS

Weights	Spearman	95% CI	*p *value*
Original	0.41	0.23–0.56	<0.0001
Weight (1)	0.43	0.26–0.58	<0.0001
(2)	0.48	0.31–0.62	<0.0001
(3)	0.44	0.27–0.59	<0.0001

Three different weighting systems were pursued: (1) exchanging the maximum scores of the clinical status subscale and the surgery subscale; (2) up-weighting the clinical status subscale by a factor of two, and removing the surgery subscale and the extra-articular manifestation subscale; (3) multiplying each subscale by the regression coefficients of a linear regression of all subscales on the DAS28. CI, confidence interval; DAS28, 28-joint Disease Activity Score; RARBIS, rheumatoid arthritis medical records-based index of severity.

## References

[B1] Grassi W, De Angelis R, Lamanna G, Cervini C (1998). The clinical features of rheumatoid arthritis. Eur J Radiol.

[B2] Lawrence RC, Helmick CG, Arnett FC, Deyo RA, Felson DT, Giannini EH, Heyse SP, Hirsch R, Hochberg MC, Hunder GG (1998). Estimates of the prevalence of arthritis and selected musculoskeletal disorders in the United States. Arthritis Rheum.

[B3] Scott DL, Symmons DP, Coulton BL, Popert AJ (1987). Long-term outcome of treating rheumatoid arthritis: results after 20 years. Lancet.

[B4] Pincus T, Brooks RH, Callahan LF (1994). Prediction of long-term mortality in patients with rheumatoid arthritis according to simple questionnaire and joint count measures. Ann Intern Med.

[B5] Wolfe F, Mitchell DM, Sibley JT, Fries JF, Bloch DA, Williams CA, Spitz PW, Haga M, Kleinheksel SM, Cathey MA (1994). The mortality of rheumatoid arthritis. Arthritis Rheum.

[B6] Solomon DH, Karlson EW, Rimm EB, Cannuscio CC, Mandl LA, Manson JE, Stampfer MJ, Curhan GC (2003). Cardiovascular morbidity and mortality in women diagnosed with rheumatoid arthritis. Circulation.

[B7] Gridley G, McLaughlin JK, Ekbom A, Klareskog L, Adami HO, Hacker DG, Hoover R, Fraumeni JF (1993). Incidence of cancer among patients with rheumatoid arthritis. J Natl Cancer Inst.

[B8] Lee JH, Slifman NR, Gershon SK, Edwards ET, Schwieterman WD, Siegel JN, Wise RP, Brown SL, Udall JN, Braun MM (2002). Life-threatening histoplasmosis complicating immunotherapy with tumor necrosis factor alpha antagonists infliximab and etanercept. Arthritis Rheum.

[B9] Keane J, Gershon S, Wise RP, Mirabile-Levens E, Kasznica J, Schwieterman WD, Siegel JN, Braun MM (2001). Tuberculosis associated with infliximab, a tumor necrosis factor alpha-neutralizing agent. N Engl J Med.

[B10] Brown SL, Greene MH, Gershon SK, Edwards ET, Braun MM (2002). Tumor necrosis factor antagonist therapy and lymphoma development: twenty-six cases reported to the Food and Drug Administration. Arthritis Rheum.

[B11] Mohan N, Edwards ET, Cupps TR, Oliverio PJ, Sandberg G, Crayton H, Richert JR, Siegel JN (2001). Demyelination occurring during anti-tumor necrosis factor alpha therapy for inflammatory arthritides. Arthritis Rheum.

[B12] Matteson EL, Hickey AR, Maguire L, Tilson HH, Urowitz MB (1991). Occurrence of neoplasia in patients with rheumatoid arthritis enrolled in a DMARD Registry. Rheumatoid Arthritis Azathioprine Registry Steering Committee. J Rheumatol.

[B13] Silman AJ, Petrie J, Hazleman B, Evans SJ (1988). Lymphoproliferative cancer and other malignancy in patients with rheumatoid arthritis treated with azathioprine: a 20 year follow up study. Ann Rheum Dis.

[B14] Walker AM (1996). Confounding by indication. Epidemiology.

[B15] Strom B (2000). Pharmacoepidemiology.

[B16] Cabral D, Katz JN, Weinblatt ME, Ting G, Avorn J, Solomon DH (2005). Development and assessment of indicators of rheumatoid arthritis severity: results of a Delphi panel. Arthritis Rheum.

[B17] Ting G, Schneeweiss S, Katz JN, Weinblatt ME, Cabral D, Scranton RE, Solomon DH (2005). Performance of a rheumatoid arthritis records-based index of severity. J Rheumatol.

[B18] van der Heijde DM, van't Hof M, van Riel PL, van de Putte LB (1993). Development of a disease activity score based on judgment in clinical practice by rheumatologists. J Rheumatol.

[B19] Prevoo ML, van't Hof MA, Kuper HH, van Leeuwen MA, van de Putte LB, van Riel PL (1995). Modified disease activity scores that include twenty-eight-joint counts. Development and validation in a prospective longitudinal study of patients with rheumatoid arthritis. Arthritis Rheum.

[B20] Wolfe F (1997). The prognosis of rheumatoid arthritis: assessment of disease activity and disease severity in the clinic. Am J Med.

[B21] Wolfe F, O'Dell JR, Kavanaugh A, Wilske K, Pincus T (2001). Evaluating severity and status in rheumatoid arthritis. J Rheumatol.

[B22] Symmons DP, Hassell AB, Gunatillaka KA, Jones PJ, Schollum J, Dawes PT (1995). Development and preliminary assessment of a simple measure of overall status in rheumatoid arthritis (OSRA) for routine clinical use. QJM.

[B23] Birrell FN, Hassell AB, Jones PW, Dawes PT (1998). Why not use OSRA? A comparison of Overall Status in Rheumatoid Arthritis (RA) with ACR core set and other indices of disease activity in RA. J Rheumatol.

[B24] Bardwell WA, Nicassio PM, Weisman MH, Gevirtz R, Bazzo D (2002). Rheumatoid Arthritis Severity Scale: a brief, physician-completed scale not confounded by patient self-report of psychological functioning. Rheumatology (Oxford).

[B25] Navarro-Cano G, Del Rincon I, Pogosian S, Roldan JF, Escalante A (2003). Association of mortality with disease severity in rheumatoid arthritis, independent of comorbidity. Arthritis Rheum.

[B26] Hochberg MC, Change RW, Dwosh I, Lindsey S, Pincus T, Wolfe F (1992). The American College of Rheumatology revised criteria for the classification of global functional status in rheumatoid arthritis. Arthritis Rheum.

